# Comparison of Pericardiocentesis in Post-Cardiac Surgery and Nonsurgical Patients with Pericardial Tamponade

**DOI:** 10.21470/1678-9741-2020-0714

**Published:** 2022

**Authors:** Aleks Değirmencioğlu, Gültekin Karakuş, Ertuğrul Zencirci, Ahmet Ümit Güllü, Şahin Şenay

**Affiliations:** 1 Department of Cardiology, School of Medicine, Acibadem University, Istanbul, Turkey.; 2 Department of Cardiovascular Surgery, School of Medicine, Acibadem University, Istanbul, Turkey.

**Keywords:** Cardiac Tamponade, Cardiovascular Surgical Procedures, Pericardiocentesis, Punctures, Drainage

## Abstract

**Introduction:**

There are several approaches for pericardiocentesis. However, there is no definite suggestion about puncture location after cardiac surgery. The purpose of this study is to examine whether there is any difference regarding puncture location during pericardiocentesis in postoperative cardiac tamponade comparing to nonsurgical cardiac tamponade.

**Methods:**

We retrospectively analyzed patients who had undergone pericardiocentesis from August 2011 to December 2019. Patients were examined in two groups, nonsurgical and postsurgical, based on the etiology of pericardial tamponade. Clinical profiles, echocardiographic findings, and procedural outcomes were identified and compared.

**Results:**

Sixty-eight pericardiocenteses were performed in this period. The etiology of pericardial effusion was cardiac surgery in 27 cases and nonsurgical medical conditions in 41 cases. Baseline demographic variables were similar between the surgical and nonsurgical groups. Loculated effusion was more common in the postsurgical group (48.1% *vs*. 4.9%, *P*<0.001). Maximal fluid locations were different between the groups; right ventricular location was more common in the nonsurgical group (36.6% *vs*. 11.1%, *P*=0.02), while lateral location was more common in the postsurgical group (12.2% *vs*. 40.7%, *P*=0.007). Apical drainage was more frequently performed in the postsurgical group compared to the nonsurgical group (77.8% *vs*. 53.7%, *P*=0.044).

**Conclusion:**

Apical approach as a puncture location can be used more frequently than subxiphoid approach for effusions occurred after cardiac surgery compared to nonsurgical effusions. Procedural success is prominent in this group and can be the first choice of treatment.

**Table:** 

Abbreviations, acronyms & symbols	
CRP	= C-reactive protein
RV	= Right ventricular

## INTRODUCTION

Pericardial tamponade is a life-threatening condition and pericardiocentesis is a lifesaving procedure. Subxiphoid approach has been preferred because it was considered the safest route without echocardiographic imaging guidance. However, there is no definite suggestion about puncture location in the era of echocardiography-guided pericardiocentesis, and there are important studies using different entry sites^[1^-^4]^.

Echocardiographic examination is very important to assess the distribution and amount of pericardial effusion because it is not always circumferential and equally distributed. Imaging allows defining the clear location of the effusion, the ideal puncture site, and needle trajectory before pericardiocentesis. The ideal puncture site is the point at which the distance from skin to maximal fluid accumulation is minimized and which a straight needle trajectory avoids vital structures^[4]^. Apical, subcostal, or parasternal approach can be used for pericardiocentesis^[5]^. Loculated effusion is more frequent after cardiac surgery^[6]^, and the site of the effusion is usually posterolaterally located^[7^,^8]^, so the optimal location of drainage can be different when comparing with nonsurgical pericardial effusion.

The purposes of this study were to investigate the properties of postsurgical and nonsurgical pericardial tamponades and to examine whether there is any difference for puncture location during pericardiocentesis.

## METHODS

We retrospectively analyzed patients who had undergone pericardiocentesis due to pericardial tamponade from August 2011 to December 2019. Demographic characteristics, comorbid conditions, preoperative echocardiography, procedure details, and follow-up data were obtained from the hospital’s medical records. The study protocol was conducted according to the principles of the Declaration of Helsinki (2013). Informed patient consent and institutional approval (2020-18) were obtained for the study.

The indication for the procedure was cardiac tamponade in all patients, and diagnosis of cardiac tamponade was made by clinical and echocardiographic findings. Exclusion criteria for pericardiocentesis were posterior or right atrial loculation of effusion, intrapericardial clot or hematoma, suspicion of ongoing bleeding, and recurrent effusion due to malignancy.

The patients were divided into two groups according to the etiology of pericardial effusion. Postsurgical tamponade was defined as cardiac tamponade developing after cardiac surgery, and nonsurgical tamponade was defined as cardiac tamponade due to other medical conditions. Maximal fluid locations and the ideal site of needle entry were determined by echocardiography in supine position with identification of the point at which the largest fluid accumulation is closest to the skin and needle trajectory avoids vital structures^[4]^.

Successful pericardiocentesis was described as sufficient fluid drainage which resolves the clinical and echocardiographic findings of pericardial tamponade. It was performed using appropriate spatial orientation of the needle in x, y, and z axes using preoperative echocardiography without simultaneous visualization of the needle tip during procedure as we described^[9]^. After local anesthesia and mild sedation, an 18-gauge (7 cm) needle with an attached saline filled syringe was introduced from predetermined location. The superior margin of the rib was selected to avoid vascular bundle if apical location was used. When the pericardial sac was entered, the syringe was removed from the needle, and appearance of the fluid was assessed. Saline contrast injection with echocardiographic monitoring were carried out if the draining fluid was bloody to confirm the needle’s tip in the pericardial sac. 8F (15 cm) special multiple-hole catheter or pigtail catheter was inserted, and pericardial fluid was aspirated with syringe up to 500 mL initially to prevent acute right ventricular dilation and ensuing hypotension. The catheter was connected to a closed system for further drainage and was left in pericardial space until insignificant amount of effusion was seen during echocardiographic follow-up.

### Statistical Analysis

Quantitative variables were expressed as mean (± standard deviation), and qualitative variables were expressed as percentage (%). Data were tested for normal distribution using the Kolmogorov-Smirnov test. A comparison of parametric values between two groups was made using a two-tailed Student t-test, and Mann-Whitney U test was used for nonparametric values. Categorical variables were compared using the chi-squared test or Fisher’s test. A *P*-value of <0.05 was considered statistically significant. All statistical analyses were carried out using SPSS Inc. Released 2009, PASW Statistics for Windows, version 18.0, Chicago: SPSS Inc.

## RESULTS

Between August 2011 and December 2019, 68 pericardiocenteses were performed. The etiology of pericardial effusion was cardiac surgery in 27 cases and nonsurgical medical conditions in 41 cases. A total of 2,450 heart surgeries were performed in this period, and the incidence of pericardial tamponade requiring pericardiocentesis following cardiac surgery was 1.1%. Four patients were not included in the study because surgical drainage was performed in these patients instead of pericardiocentesis due to right atrial loculation of the effusion (two patients), suspicion of ongoing bleeding (one patient), and recurrent effusion due to malignancy (one patient).

Table 1 shows the baseline demographic, echocardiographic, and laboratory characteristics of both groups. In the surgical group, 21 procedures (77.8%) were performed in male patients and their average age was 54.7±11.4 years. There were no significant differences between age, gender distribution, maximal fluid thickness, hemoglobin, and C-reactive protein levels between the groups. Usage of anticoagulant treatment was more common in the surgical group (59.3% in the postsurgical group *vs*. 0% in the nonsurgical group; *P*<0.001). Loculated effusion was also more common in the postsurgical group (48.1% in the postsurgical group *vs*. 4.9% in the nonsurgical group; *P*<0.001). Maximal fluid locations were different between the groups. Right ventricular location was more common in the nonsurgical group (11.1% in the postsurgical group *vs*. 36.6% in the nonsurgical group; *P*=0.02), whereas lateral location was more common in the postsurgical group (40.7% in the postsurgical group *vs*. 12.2% in the nonsurgical group; *P*=0.007). Apical location was similar between the groups (48.1% in the postsurgical group *vs*. 51.2% in the nonsurgical group; *P*=0.8).

**Table 1:** Baseline clinic, echocardiographic, and laboratory characteristics of patients.

	Nonsurgical group (n= 41)	Postsurgical group (n=27)	*P*-value
Age (years)	54.9±17.1	54.7±11.4	0.96
Male [n, (%)]	24 (58.5)	21 (77.8)	0.1
Warfarin [n, (%)]	0 (0)	16 (59.3)	< 0.001
Fluid distribution (loculated/circumferential) [n, (%)]	2 (4.9)/39 (95.1)	13 (48.1)/14 (51.9)	< 0.001
Maximal fluid thickness (mm)	30.7±9.7	33.8±8.2	0.21
Maximal fluid location (apical/lateral/RV) [n, (%)]	21 (51.2)/5 (12.2)/15 (36,6)	13 (48.1)/11 (40.7)/3 (11.1)	0.8/0.007/0.02
CRP	9.4±6.1	6.2±3.2	0.06
Hemoglobin (g/dl)	11.1±2.1	10.5±2.2	0.25

CRP=C-reactive protein; RV=right ventricular

Procedural outcomes are shown in Table 2. Subxiphoid and apical approaches were used for drainage. Successful pericardial drainage was performed in all patients in the postsurgical group who had been selected for pericardiocentesis without any complication or need for any surgical drainage. Apical approach (n=21) was performed more frequently than subxiphoid approach (n=6) in the postsurgical group, while frequency of both approaches was similar in the nonsurgical group (apical=22, subxiphoid=19). Apical approach was statistically significantly more common in the postsurgical group compared to the nonsurgical group (*P*=0.044).

**Table 2:** Procedural outcomes.

	Non-surgical group (n=41)	Postsurgical group (n=27)	*P*-value
Drainage location (apical/subxiphoid) [(n, %)]	22 (53.7)/19 (46.3)	21 (77.8)/6 (22.2)	0.044
Procedural success (n, %)	39 (95.1)	27 (100)	0.51
Appearance of effusion (serous/serohemorrhagic/hemorrhagic) [(n, %)]	16 (39)/7 (17.1)/18 (43.9)	5 (18.5)/9 (33.3)/13 (48.2)	0.07/0.12/0.73
Drainage volume (ml)	974.9 ± 894.8	748.8 ± 333.2	0.52
Duration of drainage (hour)	33.1 ± 48.9	19.9 ± 19.9	0.21
Minor complication (n, %)	5 (12.2)	0 (0)	0.15
Recurrence (n, %)	4 (9.8)	3 (11.1)	0.99
Emergency procedure (n, %)	10 (24.4)	5 (18.5)	0.57
In-hospital mortality (n, %)	5 (12.2)	0 (0)	0.15

In the nonsurgical group, pericardiocentesis could not be performed in two patients due to dense fibrinous effusion, and surgical evacuation was performed. Minor complications occurred in five patients and repeat pericardiocentesis was required in one patient who had also pleural effusion, because puncture and drainage of pleural space was performed on first attempt. There were no statistically significant differences in procedural success and complication rate between the two groups.

Recurrence rates were also similar between the groups; and surgical drainage was performed in one patient in the nonsurgical group, and close follow-up was performed in another three patients. Three patients undergone second pericardiocentesis in surgical group.

Mean duration of drainage was 19.9±19.9 hours and mean drainage volume was 748,8±333,2 ml in the surgical group. The mean time from surgery to diagnosis of tamponade and pericardiocentesis was 29.3±23.1 days. The character of pericardial effusions was serous in 18.5% *vs*. 39% (*P*=0.07), serohemorrhagic in 33.3% *vs*.17.1% (*P*=0.12), and hemorrhagic in 48.2% *vs*. 43.9% (*P*=0.73) of the patients in the surgical and nonsurgical groups, respectively. Pericardial tamponade and the need for pericardiocentesis was more common after valve surgery than coronary artery bypass grafting (16 *vs*. 9 patients). Most frequent etiologies were malignancy (25 patients) and infection (11 patients) in the nonsurgical group. Five patients died related to underlying malignancy despite successful pericardiocentesis in the nonsurgical group during index hospitalization while no mortality was seen in the surgical group. No significant difference was observed in drainage volume, duration of drainage, character of fluid, and in-hospital mortality rate between the two groups (*P*>0.05)

## DISCUSSION

Any pathology that leads to inflammation, injury, or decreasing lymphatic drainage of the pericardium can result in a pericardial effusion^[10]^. Cardiac surgery is one of the most important reasons for pericardial effusion and, occasionally, it may lead to cardiac tamponade requiring immediate drainage^[11]^. Reported incidence of postoperative pericardial effusion which necessitates drainage is 1% to 2%^[6^,^12]^, similar to our finding.

Pericardiocentesis is a lifesaving procedure in the presence of cardiac tamponade in most cases and is generally preferred to surgical drainage because it is less invasive and a more comfortable approach for the patients. It is also the procedure of choice in unstable patients because it can be performed more quickly at the bedside, and patients with tamponade can deteriorate with induction of anesthesia during surgical drainage. It is especially safe and effective in the treatment of circumferential or anterior effusions, however, some patients may need surgical drainage^[13]^. Patients with posterolateral loculation of effusion, isolated effusion along the right atrial wall, intrapericardial clot or hematoma, suspicion of ongoing bleeding, and multiple intrapericardial echoes are potential candidates for surgical drainage^[14]^, and these pathologies are more frequent in effusions developed following cardiac surgery^[8^,^15]^. In addition, surgical drainage is preferred in patients with recurrent effusions and in patients who need pericardial biopsy for diagnosis.

There are three main approaches for pericardiocentesis: subxiphoid approach, apical approach, and, rarely, parasternal approach. Traditionally, subxiphoid approach was most commonly used^[16]^. If the effusion is distributed circumferentially around the heart, subxiphoid approach is generally preferred^[14]^. However, if it is nonuniform or localized, the site of maximum accumulation of effusion closest to the skin should be used for pericardiocentesis^[14]^. One study revealed that postoperative cardiac tamponade was treated effectively by subxiphoid puncture when the effusion thickness was at least 10 mm from subcostal echocardiographic window, however, more localized, apical, or laterally located effusions could not be treated percutaneously in this study^[8]^. There are also some important studies in which subxiphoid approach was used predominantly^[2^,^17^-^19]^. However, some series demonstrated a higher successful rate and lower complication rate when the entry site was selected echocardiographically instead of the routine subxiphoid approach^[4^,^20]^. Tsang et al.^[3^,^15]^ reported two large retrospective series, being one of them related with postsurgical effusion, and they performed the procedure with apical approach more frequently than subxiphoid approach.

In this study, we investigated retrospectively general properties and the drainage location of postoperative cardiac tamponade and compared with nonsurgical cardiac tamponade. Baseline patient characteristics were similar between the groups, except frequency of warfarin usage and the type of fluid distribution. Much more apparent usage of warfarin is due to frequent valvular surgery in the surgical group. It was reported that the incidence of large effusions were significantly higher in patients who received anticoagulation^[21]^, and postoperative cardiac tamponade appears to be more common following valve surgery than coronary artery bypass graft surgery^[22]^, similar to our finding. Also, postsurgical effusions are more frequently loculated especially in the posterolateral side of the left ventricle, similar to our finding^[6^,^8^,^15]^. On the other side, right ventricular location was found more common in nonsurgical effusions in our study, and this can be explained by more frequent occurrence of circumferential effusion in these patients, because maximum accumulation is influenced by the patient supine position.

While the subxiphoid approach is safe and the most practiced approach with minimal complications in circumferential effusions, this technique cannot be used if the effusion is localized and absent in the right and inferior part of the pericardium because there would be risk of cardiac perforation^[23]^, so apical puncture is an important alternative location for pericardiocentesis in these patients by reaching the lateral side of the left ventricle. Successful pericardiocentesis was performed in all patients in the postsurgical group without any complication or need for any surgical drainage. More common using of apical approach in our postsurgical patients supports that apical approach is more valuable in patients with postoperative cardiac tamponade and this seems to be related with different location of postsurgical effusion compared to nonsurgical effusion.

### Limitations

The main limitation of this study is its retrospective nature, we obtained all information from our medical records. This series represents the experience of a single center with a limited number of patients. Additionally, experience of the operator is also important to plan the treatment and it may influence the decision. Therefore, we could not be sure that apical approach is a possible and safe approach in every case after cardiac operation.

## CONCLUSION

Apical and subxiphoid regions are important puncture locations for pericardiocentesis and they could be used nearly equally in nonsurgical pericardial tamponade. However, postsurgical pericardial effusions, which may be localized posterolaterally and spread to the anterolateral part of the left ventricle, could be drained more frequently with apical approach. Thus, apical approach is a possible important option for the treatment of postsurgical pericardial tamponade and it may decrease the need for surgical intervention.

**Table:** 

Authors' roles & responsibilities	
AD	Substantial contributions to the conception and design of the work; and the acquisition, analysis, and interpretation of data for the work; final approval of the version to be published
GK	Substantial contributions to the conception and design of the work; and the analysis and interpretation of data for the work; final approval of the version to be published
EZ	Substantial contributions to the acquisition and analysis of data for the work; final approval of the version to be published
AÜG	Substantial contributions to the acquisition of data for the work; final approval of the version to be published
ŞŞ	Revising the work; final approval of the version to be published
